# Paralization and online adaptation of an ongoing clinical trial in La Paz University Hospital (Madrid, Spain) during the COVID-19 worldwide pandemic

**DOI:** 10.1192/j.eurpsy.2022.1582

**Published:** 2022-09-01

**Authors:** M.P. Vidal-Villegas, J. Andreo Jover, R. Mediavilla, A. Muñoz-Sanjosé, B. Rodríguez-Vega, C. Bayón-Pérez, Á. Palao Tarrero, M.F. Bravo-Ortiz

**Affiliations:** 1 Hospital La Paz Institute for Health Research (IdiPAZ), Psychiatry And Mental Health, Madrid, Spain; 2 Autonomous University of Madrid, Department Of Psychiatry, Madrid, Spain; 3 Centro de Investigación Biomédica en Red de Salud Mental, Cibersam, Madrid, Spain; 4 La Paz University Hospital, Psychiatry, Clinical Psychology And Mental Health, Madrid, Spain

**Keywords:** healthcare psychotherapy clinical trial

## Abstract

**Introduction:**

Spain went into lockdown in March of 2020 due to the COVID-19 outbreak. We had to stop the third randomization of our ongoing clinical trial (Mediavilla et al., 2019), pausing weekly group psychotherapy for 12 people with a first episode of psychosis. Only 5 weekly sessions had been delivered, thus many were just starting to form a therapeutic link with the group. In a public health emergency context, psychotherapeutic groups are considered avoidable gatherings. However, stopping psychological therapy abruptly can make participants more vulnerable. The intervention groups were launched in an online format because we could not let anyone go without psychological support in such a difficult time.

**Objectives:**

Communicate how we adapted an ongoing clinical trial to an online format during the lockdown in Spain.

**Methods:**

In light of our participants’ needs and their acute deterioration the first two weeks of lockdown, we adapted our intervention. First, both arms (mindfulness-based v. psychoeducational multicomponent intervention) began online adaptations of the interventions. Second, a research assistant made weekly phone calls to provide basic psychosocial support, assure participants groups would continue, and later remind them of each online session. Third and last, the phoneline was accessible 24/7 (WhatsApp).

**Results:**

The third randomization concluded in December. Six participants were lost in the transition to online groups. However, adherence was comparable to the previous two randomizations (4/12 completed the intervention).

**Conclusions:**

Online psychotherapy may be used in emergencies such as a lockdown. However, the psychological mid- and long-term effects of a lockdown and online group therapy remain unknown.

**Disclosure:**

No significant relationships.

